# Short-interference RNAs: becoming medicines

**DOI:** 10.17179/excli2015-297

**Published:** 2015-06-15

**Authors:** Tamara Martínez, Ana Isabel Jiménez, Covadonga Pañeda

**Affiliations:** 1 Sylentis, R&D department c/Santiago Grisolía, Tres Cantos, Madrid, Spain

**Keywords:** siRNA, RNAi, oligonucleotides, therapeutics

## Abstract

RNA interference is a cellular mechanism by which small molecules of double stranded RNA modulate gene expression acting on the concentration and/or availability of a given messenger RNA. Almost 10 years after Fire and Mello received the Nobel Prize for the discovery of this mechanism in flat worms, RNA interference is on the edge of becoming a new class of therapeutics. With various phase III studies underway, the following years will determine whether RNAi-therapeutics can rise up to the challenge and become mainstream medicines. The present review gives a thorough overview of the current status of this technology focusing on the path to the clinic of this new class of compounds.

## RNA Interference: Mechanism of Action

RNA interference (RNAi) is an evolutionary conserved, endogenous process for post-transcriptional regulation of gene expression. Since the discovery of RNAi in *C. elegans* the mechanistic details of the RNAi pathway have been largely characterized. The discovery of RNAi fueled an extensive research aimed towards the application of this novel tool for therapeutic purposes as well as for further understanding of the mechanisms of gene regulation. These investigations led to the identification of short sequences of double-stranded RNAs (dsRNAs) as molecular mediators of RNAi (Fire et al*.*, 1998[44]). With the 2001 report by Elbashir and collaborators[40], showing that 21-mer dsRNAs were able to mediate specific gene silencing in mammalian cells *in vitro*, the basic mechanism of RNAi became available, allowing the initiation of a new era in manipulation of gene expression. This new technology, that could potentially silence any gene of interest, was immediately considered the basis for a revolutionary new class of drugs, with applications in numerous diseases, where deregulation of gene expression contributes to pathogenesis. Eukaryotic cells are able to modulate gene expression by transcribing RNAs that use the RNAi machinery to silence or repress the expression of specific genes. These modulators of gene expression are transcribed from non-coding regions of the genome yielding micro-RNAs (miRNAS) or short interfering-RNAs (siRNAs). siRNAs are transcribed as long dsRNAs that are transported into the cytoplasm and cleaved by DICER to yield siRNAs of 19 to 24 nucleotides with phosphorylated 5'-ends and hydroxylated 3'-ends. The cleaved RNAs are subsequently incorporated into a multiprotein complex named RNA-induced silencing complex (RISC). Using the thermodynamic stability of the ends as the main criteria, the RISC incorporates one of the strands known as the guide or antisense strand (AS) into the complex; while the other strand, known as the passenger or sense strand (SS), is discarded and degraded (Schwarz et al., 2003[118]). The proteins of the RISC facilitate the correct spatial orientation of the antisense strand in order to expose the seed region (nucleotides 2 to 8 from the 5' end). The seed region is then used by the complex to locate mRNAs that have sequences that are complementary or partially complementary to the seed region. When perfectly complementary sequences are located the argonaute 2 (AGO2) protein of the RISC promotes degradation of the mRNA, thus blocking translation. Partial match between the mRNAs and the seed region cause translational repression rather than degradation of the mRNA. miRNAs are transcribed from endogenous mi-RNA genes as primary miRNAs (pri-miRNAs) containing 60-70 nucleotide stem loop structures. These long molecules are processed in the nucleus into precursory miRNAs (pre-miRNAs) by DROSHA/DGCR8 and are thereafter transported from the nucleus to the cytoplasm by exportin 5. In the cytoplasm, pre-miRNAs are further cleaved by Dicer to yield a 20-23bp long mature miRNA. Mature miRNAs are loaded into the RISC to elicit translational inhibition with target mRNA degradation or sequestration in cytoplasmic P-bodies (Bartel, 2004[8]). Unlike siRNAs and shRNAs, miRNAs are only partially complementary to their target mRNA 3' UTR region, regulating their targets via all four AGO proteins (AGO1-4). In this case perfect match is not necessary for miRNA to function, thus the change in the expression of a single RNA may affect hundreds of genes (John et al., 2004[60]). siRNAs and miRNAs can be exogenously incorporated into cells to engage RNAi; this principle is the basis for the use of small RNAs for therapeutic purposes (Elbashir et al., 2001[40]; Martinez et al., 2002[87]). By far, the most popular siRNA design mimics natural Dicer cleavage products. These products have the so called canonical design and comprise a 21 nucleotide guide strand antisense and a complementary passenger strand annealed to form a siRNA duplex with a 19-bp dsRNA stem and 2 nucleotide 3′ overhangs at both ends (Elbashir et al., 2001[40][41]). The canonical design can be modified to make it shorter or truncated. Examples of these designs include 16-mer siRNAs (Chu and Rana, 2008[27]), blunt 19-bp siRNAs (Czauderna et al., 2003[32]), asymmetrical siRNAs (Sun et al., 2008[129]) and asymmetric shorter-duplex siRNA (asiRNA) (Chang et al., 2009[22]).

shRNAs are expressed in cells by delivery of plasmids or through viral or bacterial vectors, (Xiang et al., 2006[141]; Lombardo et al., 2007[82]) and are transcribed in the nucleus from an external expression vector bearing a short-doubled stranded DNA sequence with a hairpin loop by a RNA polymerase II or III. The shRNA transcript is then processed by DROSHA, a RNAse III endonuclease. The resulting pre-shRNA is exported to the cytoplasm, where it is processed by Dicer into 21-25 nt small RNAs, and incorporated into RISC were it exerts its action (Yu et al*.*, 2002[144]).

The present review focuses on recent advances relevant to the use of siRNAs as therapeutic tools. In the following sections we will first address general challenges associated with the use of siRNAs as drugs. We will also discuss in detail different strategies used to improve the pharmaceutical properties of therapeutic siRNAs; such as chemical modifications, delivery strategies as well as the key elements that should be taken into account when designing siRNA-based compounds. Finally we will give a brief update on preclinical and clinical developmental programs highlighting the specific contributions of each of these programs to the field of RNAi therapeutics. 

## RNAi Therapeutics

Traditional pharmaceuticals or “small molecules” typically tackle diseases by targeting proteins. Most small molecules target membrane proteins and/or receptors that are readily available to bind to these agents. This means that a whole range of targets, mostly present in the cytoplasm or nucleus of cells, are not available for traditional targeting. In contrast to small molecules, siRNAs can be designed to target virtually any gene; including the traditionally “undruggable” targets. On the other side, the avenue primed by discoveries in the genetics field in the last decade has significantly boosted the identification of new drug targets for diseases with previously unknown subjacent mechanisms. Observational human genetic studies have significantly contributed to the validation of targets for a given condition (Plenge et al., 2013[108]) facilitating the way for target-directed therapeutics that can take advantage of this accumulated knowledge. 

RNAi based compounds are chemically synthetized and therefore considered New Chemical Entities (NCEs) from a regulatory standpoint; however some of the characteristic features of these compounds are closer to those of New Biological Entities (NBE). One of these characteristics is an enormous specificity for the target against which they are designed. The specificity displayed by siRNAs to recognize their target enables the design of compounds to target dysfunctional proteins encoded by genes with single point mutations in one allele, hence opening a new path for the treatment of genetic disorders (Martinez et al*.*, 2013[89]). In addition, exogenously administered unmodified siRNAs are identical to those synthetized by cell transcription and processing, therefore their breakdown products can be incorporated to the endogenous pathways of degradation of other oligonucleotides. Compared to other oligonucleotide based therapeutic options, strategies based on RNAi achieve greater efficacy since a single guide strand can be used for several rounds of mRNA cleavage providing a beneficial effect at lower concentrations (Bertrand et al*.*, 2002[11]). This has broad implications in the clinical setting, as it would mean less frequent and/or lower doses, thus decreasing the risk of unwanted side effects. Finally, siRNA-based drug candidates are designed using bioinformatic tools to select sequences complementary to the target mRNA, thereafter algorithms are used to select the specific candidates with the highest likelihood of efficacy in humans. In addition, candidates can usually be designed to cross-react with the homologous gene of other animal species ensuring activity in the animal model that will be used for efficacy or toxicology testing. This latter characteristic is thought to simplify translation to humans from animal models. 

Despite the advantages and the great therapeutic potential of RNAi-based therapies, translation of siRNAs into therapeutic applications has turned out to be a much more tedious task than originally thought and is still hindered by multiple obstacles. The *in vivo* efficacy of siRNA based agents is dependent on intact siRNAs to reach the cytoplasm of their target cell, given the size and charge of naked siRNAs this task has stemmed more complicated than anticipated (Vicentini et al*.*, 2013[135]). The following sections will go through the specific characteristics that make siRNAs a very attractive class of therapeutic agents highlighting the challenges that need to be addressed in order to mainstream their use in the clinical setting. 

## Stability of RNAi Based Therapeutics: Chemical Modification

Unmodified oligonucleotides are extremely sensible to degradation by nucleases. Nucleases cleave the phosphodiester bonds of nucleic acids and play essential roles in diverse cellular processes such as DNA repair, DNA replication and RNA splicing. Nucleases are also a fundamental part of the defense system of biological organisms (Yang, 2011[143]). Nucleases are very abundant in biological fluids and tissues; their concentration and type varies among tissues and cellular compartments (Sorrentino, 2010[125]). Nuclease activity significantly hampered development of RNAi therapeutics during its initial years; this drawback was however tackled by chemically modifying siRNAs. Various chemical modifications have been developed in order to increase stability of oligonucleotides; these modifications can be used alone or in combination to achieve specific goals. It should be kept in mind that modifications originally applied to other oligonucleotides such as antisense oligonucleotides or aptamers may not be optimal for siRNAs as the mechanism of action of these oligonucleotides significantly differs. As such, chemical modification of siRNAs should be rationalized to ensure compatibility with the RISC machinery. Tolerance for chemical modification of a given part of a siRNA is dependent on the physical and chemical characteristics of the chemical groups as well as the position where they are located. Ultimately, the activity of modified siRNAs needs to be confirmed in a biological setting to ensure silencing activity has not been lost. 

As mentioned above, modifications of the siRNA molecule must be compatible with the endogenous RNAi pathways. As a general rule, the SS and the 3'-proximal part and 3'-overhang of the AS are more amenable to chemical modification; on the contrary the 5' phosphate, the 5' proximal part and central positions of the AS are more sensitive, especially to multiple or bulky modifications (Bramsen and Kjems, 2013[17]). The 5'-phosphate group of the guide strand is required for the binding to AGO2 (Nykanen et al*.*, 2001[100]; Wang et al., 2008[136]), in addition, the initial interactions between the guide strand and target RNA are mediated by the 5' proximal seed region; therefore modifications of these particular regions may interfere with activity (Wang et al., 2008[136]). 

Oligonucleotides are synthetized using solid phase phosphoroamidite chemistry. As such, amidites are incorporated into a growing chain as building blocks to obtain a full length single strand with the adequate length and sequence. Two complementary strands are thereafter hybridized to yield a siRNA. The availability of modified phosphoroamidites significantly simplifies chemical modification of siRNAs, thus most modifications can be incorporated without significantly altering the synthesis process. 

There are three groups of modification used to improve stability of siRNAs: (1) modification of the phosphodiester backbone, (2) sugar modifications and (3) modification of the ribose ring and nucleoside bases. This review will focus on the modification used in compounds in clinical development or in compounds that are close to reach this goal.

### Modification of the phosphodiester backbone

Nucleotides composing RNA and DNA are linked to each other by phosphodiester linkages. These bonds are negatively charged at physiological pH and are easily cleaved by endo- and exonucleases. Modification of this negatively charged backbone is typically used to enhance oligonucleotide resistance against nuclease degradation. The most widely used phosphate backbone alteration is the phosphorothioate (PS) modification in which the non-bridging oxygen atom in the phosphate backbone is replaced by a sulfur atom. The PS modification introduces chirality to inter-nucleotide linkages, one of the stereochemistries provides resistance to nuclease-mediated cleavage (Eckstein, 2002[38]). Although strategies for selecting the stereochemistry of the PS linkage have been described they are usually synthetized as diastereoisomers thus increasing the overall stability of the molecule (Guga and Stec, 2003[50]). In addition, PS linkages are cost effective modifications, easily incorporated using standard solid-phase oligonucleotide synthesis protocols (Levin, 1999[77]; Sanghvi, 2011[115]). This modification is a major component of first generation antisense oligonucleotides (ASOs) and is present in the all the FDA approved oligonucleotide drugs, pegabtanib, fomivirsen and mipomersen. As such, the change in physical and chemical properties that this modification provides when included in a molecule, as well as the related toxicities are well characterized (Eckstein, 2014[39]) (see safety section of this review). Depending on the position, PS bonds can affect activity; therefore these modifications are usually combined with other modifications that provide additional serum stability in order to avoid excessive loss of activity. In addition, PS bond lower the melting temperature of siRNAs (approximately 0.5 °C per modification), thus potentially reducing specificity. For these reasons, activity needs to be confirmed in a case per case basis (Deleavey et al*.*, 2009[34]). An alternative to the PS bond is the boranophosphate inter-nucleotide linkage, which also provides chirality and is more nuclease-resistant and less toxic than PS (Hall et al*.*, 2004[51]). On the downside, boranophosphate-modified siRNAs are not easily manufactured and issues with synthesis and industrial scalability have to be overcome before they can be used on a regular basis. Other internucleotide phosphodiester linkages include N' phosphoramidate (NP) linkages, phosphoacetate linkages (PACE), morpholino phosphoramidates, and peptide nucleic acids (PNA). NP linkages are formed by substitution of the 3'-OH by 3'-amine functionality (Chen et al*.*, 1995[23]). Phosphonoacetate (PACE) linkages are formed by substituting for the non-bridging oxygen in the nucleotide linkage by acetic group; as previously seen in the PS linkages, this introduces chirality to the linkage reducing the ability of nucleases to break the bond (Sheehan et al*.*, 2003[120]). Morpholino phosphoramidites are uncharged substitutes of internucleotide phosphodiester linkages and the furanose sugars of nucleic acids (Moulton and Jiang, 2009[95]). Peptide Nucleic Acids (PNA) have a neutral charge backbone composed of N-(2-aminoethyl)-glycine (Demidov et al*.*, 1994[35]); thus they have a peptide backbone instead of the traditional sugar-phosphate backbone. Backbone modifications increase stability against nucleases of the molecules that carry them; some of them also affect the binding affinity, rate of cellular uptake and biodistribution properties.

### Sugar modifications

Modification of the sugar moiety of nucleotides is widely used with the main purpose of enhancing siRNA nuclease resistance and hybridization properties. Free residues of the sugar ring can be modified at different positions or even the whole ribose ring can be substituted for a different sugar. The most frequently modified positions of the ribose are the 2' and 4'. Structural analyses of the silencing complex have revealed specific requirements of siRNAs to be able to induce gene silencing. These studies indicated that the 2'-OH group of the ribose ring is not specifically required for the silencing activity. On the other hand, the formation of an A-helix between the mRNA and the AS strand is strictly required for activity because the RISC complex uses the major groove to recognize the mRNA-AS-strand duplex. Modifications to the 2'-OH of the sugar ring do not fall within this category but are on the other hand required for the nuclease mediated breakdown of the oligonucleotide backbone (Chiu and Rana, 2003[25]). Accordingly, the 2'-OH of the ribose ring has been heavily modified with the aim of increasing stability without significantly affecting affinity. 2'-O-methyl (2'-O-Me) and 2'-Fluoro (2'-F) are among the most widely used modifications of the 2'OH position. Both enhance siRNA nuclease resistance and duplex thermo-stability and are generally well tolerated at most positions (Amarzguioui et al*.*, 2003[4]; Czauderna et al., 2003[32]; Chiu and Rana, 2003[25]; Prakash et al., 2005[109]). Both the 2'-O-Me and the 2'-F are present in the FDA approved drug pegaptanib thus their effect on toxicity is partially characterized (Ng et al., 2006[99]). 2'-O-Me and 2'F modifications have been combined to yield a fully modified siRNA with alternate 2'-O-Me and 2'F modified nucleotides (Allerson et al., 2005[3]). This design can yield very potent and stable compounds but has to be thoroughly tested to ensure that compounds carrying this pattern of modification maintain their silencing activity (Amarzguioui et al., 2003[4]; Chiu and Rana, 2003[25]). The 2'-O-Methoxyethyl (2'-O-MOE) residue can also be introduced into the 2' position of the sugar ring. This modification is bulky and poorly tolerated in the AS strand but is usually well tolerated in the SS strand regardless of the position (Prakash et al., 2005[109]). The most explored modification apart from the 2' position is the 4' position. In fact, the carbon of the 4' position can be bound to the carbon on the 2' position by a methylene bridge generating a conformationally closed structure known as locked nucleotide analogue (LNA). The incorporation of LNAs into a siRNA results in an increase in the melting temperature of the compound and does not necessarily alter the A-helix; thus these modifications are in principle compatible with RISC activity (Lundin et al*.*, 2013[83]). In addition, binding the two carbons removes the OH at the 2' position thus decreasing the activity of nucleases and therefore increasing biological stability. Tolerance to LNA has shown to be position dependent; not all regions of the molecule tolerate the incorporation of these analogues. As such, incorporation of these analogues to the seed region is known to significantly reduce their activity (Lundin et al., 2013[83]). Other modifications that have been introduced into the 4' residue include the 4'-thioribonucleosides (4'-S). The 4'S-modification enhances nuclease stability and increases the affinity for the target mRNA. As it is the case with other modifications, tolerance to 4'-S modifications is position dependent and are better tolerated in the SS then in the AS. In addition, these modifications have shown to render synergistic effect on stability and biodistribution when combined with 2'-O-Me or 2'-F modifications (Dande et al., 2006[33]). 

The complete 5-carbon ribose sugar can be substituted for another sugar. This is the case of 2'-deoxy-2'-fluoro-beta-D-arabinonucleic acid (FANA) modified nucleotides which are based on arabinose rather than on ribose (Dowler et al., 2006[37]). This modification increases potency and stability of candidates and it is well tolerated on the SS as well as on the ends of the AS. Another option are the unlocked nucleic acids (UNAs), which are acyclic derivatives of RNA lacking the C2'-C3' bond of the RNA ribose ring. These nucleotides are frequently used to introduce flexibility into the oligonucleotide strand. Incorporation of UNA residues can reduce duplex Tm by 5-10 °C; as a consequence extensive UNA modification will not support standard siRNA strand annealing. 

### Modification of nucleoside bases

The goal pursued when modifying nucleoside bases is to increase duplex stability while maintaining and improving native base pairing recognition and hydrogen bonding. These modifications are significantly less used than sugar and backbone modifications. Examples of nucleobase modifications include 5-bromo, 5-iodo- substitutions of uracile, 2-thio, 4-thio, dyhydro and pseudouracil. A thorough review of nucleobase modifications is out of the scope of this review and can be found in the works of Peacock and coworkers (2011[106]) or Bramsen and Kjems (2011[16]).

## Delivery and Biodistribution

Delivery of siRNA into their target tissue and cellular internalization are still major challenges that limit full exploitation of RNAi therapeutics (Burnett and Rossi, 2012[20]; Juliano et al*.*, 2014[64]). siRNAs have a negatively charged backbone and a size of about 13 kDa; characteristics that limit their ability to cross cell membranes thus restricting access to their action site. Several strategies have been pursued in order to deliver siRNAs to different organs and to increase permeability of siRNAs across biological barriers. As of today, successful and clinically relevant delivery of siRNAs has only been achieved to the liver. Strategies used to reach the liver include bioconjugation and lipid nanoparticles (LNP). Partially successful strategies have also been developed for local treatment in areas such as ophthalmology and dermatology. Other therapeutic areas still need optimization in terms of delivery in order to be of significance to the therapeutic landscape. 

### Systemic administration

Pharmaceuticals administered systemically need to reach and exit the circulatory system to reach their target organ. Systemic administration of compounds is performed by means of the enteral or the parenteral route. Molecules entering the body through the enteral route need to remain stable in the adverse conditions of the gastrointestinal (GI) tract until absorption takes place. This entails resistance to an acidic environment and to enzymes present in the GI tract. Molecules absorbed by the GI lining are thereafter required to reach the circulatory system after crossing the GI epithelium. Entrance through the parenteral routes is certainly less aggressive than the enteral route but requires the compound to be stable in the extracellular space and in circulation. In addition, molecules below 60 kDa are rapidly cleared by the kidneys; in accordance to this the elimination half-life of an intravenously (IV) injected unmodified siRNA has been shown to be of approximately 6 min (Soutschek et al*.*, 2004[126]), indicating a rapid clearance from circulation. Therefore, systemic administration also requires avoidance of excessive kidney clearance in order to increase the amount of compound reaching the target organs. Biodistribution of systemically administered naked siRNAs does not correlate with the level of organ vascularization but is mostly related to the type of endothelium lining present in the vasculature of the target organ. The liver possesses a specialized kind of endothelial cells named liver sinusoidal endothelial cells that line the capillaries. The endothelium of these capillaries has open pores lacking a diaphragm and a basal lamina underneath the endothelium allowing the passive entrance of substances (Wisse, 1970[138]). These particular characteristics make the liver an excellent target for RNAi based therapeutics. 

Once a given RNAi therapeutic has reached its target cell it needs to reach the cytoplasm where it can exert its action. The main entrance route of oligonucleotides to reach the cytoplasm is the use of one of several endocytotic pathways. Thereafter, the molecule is required to escape from the endosome before the action of enzymes and the acidification that takes place during endosome maturation degrades the compound (Juliano et al., 2014[64]). 

The major route of excretion of naked oligonucleotides is the kidney and to some extent the liver. As mentioned above siRNAs fall below the size threshold for renal filtration, accordingly, 70 % of an IV injected dose of a siRNA is found in the bladder 60 minutes after administration; most of the remaining amount of compound is cleared from circulation by the liver (Bartlett et al*.*, 2007[9]).

Biodistribution patterns observed following systemic administration of RNAi mediators indicate that their pharmaceutical properties need to be improved in order to be of value as therapeutic agents. Stability against nucleases can somewhat be overcome by chemically modifying the molecule. Other issues such as reduction of kidney clearance; increased uptake by target organs, optimization of cellular uptake and endosomal escape need however to be addressed by formulation. 

#### Strategies used for delivery to the liver

Rozema and coworkers (Rozema et al., 2007[112]) elegantly demonstrated that the combination of several strategies can yield a very effective formulation for delivering siRNAs to hepatocytes (Maier, et al., 2013[84]; Nair et al., 2014[97]). The components of this formulation consist of an endosmolytic polymer composed of butyl and amino vinyl ether (PBAVE) reversely modified with carboxy dimethylmaleic anhydride (CMD) containing PEG and N-acetilgalactosamine (NAG), together named as dynamic polyconjugate (DPC), and a cholesterol conjugated siRNA (chol-siRNA). The amphipathic and endosmolytic polymer used for the DPC is charge-masked so that the membrane activity of the polymer is only revealed in the acidic environment of the endosome. The other essential component of the DPC is the NAG attached to the masking agent that provides hepatocyte targeting via the ASGPR. NAG residues have high affinity for the ASGPR abundantly present in the plasma membrane of hepatocytes. Previous studies indicated that biodistribution of siRNAs could be greatly improved by conjugating cholesterol to the 3' end of the SS of a siRNA using a pyrrolidine linker (Soutschek et al., 2004[126]). Cholesterol conjugation provides greater binding capacity to serum proteins thus reducing kidney clearance of the siRNA, in addition cholesterol conjugation seems to improve the uptake of the siRNA by hepatocytes in a mechanism partially mediated by the low density lipoprotein receptor (LDLr). As a consequence, the elimination half-life of an intravenously administered siRNA to rats increased from 6 to 95 minutes if the siRNA was conjugated to cholesterol. Co-administering the chol-siRNA with the DPC formulation increased the efficacy of the compound up to 500-fold (Wong et al., 2012[139]). It should be mentioned that this formulation does not require the DPC to be linked to the chol-siRNA making the manufacturing process significantly simpler than if both elements of the formulation had to be covalently linked. In addition, removing the constraint of chol-siRNA attachment to the DPC has allowed for the use of other DPCs composed of different polymers and with different targeting agents (Wong et al*.*, 2012[139]). 

Lipids have been used for decades to improve the penetrance of nucleic acid into cells. This feature, together with the ability of lipids to spontaneously form vesicles, makes them exceptional carriers for oligonucleotides. Studies correlating structural properties of lipids with specific activities have allowed rational design of complex LNPs composed of lipids and other constituents in order to maximize in vivo delivery (Morrissey et al., 2005[94]; Maier et al., 2013[84]). Encapsulation of siRNAs within LNPs protects the molecules against nuclease degradation and enlarges the compound enough to avoid excessive renal filtration enabling intravenous administration. In addition, intravenously administered LNPs bind and exchange components with serum proteins; this feature can be taken advantage of for targeting purposes. The nature of the lipid component of LNPs significantly affects its properties; and consequently its biodistribution, cellular uptake rate and capacity to bind to serum proteins. Positively charged lipids facilitate interaction with negatively charged cell surfaces but increase systemic clearance whereas neutral lipids improve the biodistribution properties of the LNPs but are poorly internalized by cells. Therefore efforts to produce a rationally designed LNP have yielded particles composed of amphipathic and structural lipids whose positive charge is shielded by polyethylen glycol (PEG) conjugated lipids. The length of the PEG conjugated lipids has been finely tuned to ensure time-dependent disassociation of the PEG moiety from the LNP in blood in order to improve the transfection properties of the particle once it reaches its target cell. As a result of LNP encapsulation the serum half-life of a chemically stabilized siRNA administered intravenously to mice was improved by 8 fold (from 49 min to 6.5 h) (Morrissey et al., 2005[94]). The interaction of some of these LNPs with serum components has been thoroughly studied; the results of these studies indicate that binding of the LNP to apopoliprotein E (ApoE) significantly improves hepatocyte uptake of the LNPs via receptors present in the hepatocyte membrane such as the low density lipoprotein receptor (LDLR). Akinc and coworkers (2010[2]) demonstrated that administration of siRNA loaded ionizable LNPs to apoE^-/-^ mice significantly reduced the silencing efficacy of otherwise active siRNA loaded LNPs. These researchers also showed that the requirement of an endogenous delivery system such as the ApoE mediated hepatocyte uptake can be substituted by an exogenous targeting system. To demonstrate this phenomenon trivalent NAG residues were linked to the PEG moieties that shield the LNP. Administration of these exogenously targeted LNPs to apoE^-/-^ showed a NAG dose-dependent improvement in activity (Akinc et al., 2010[2]) .

A similar approach has been used by Nair and coworkers (2014[97]) to solve delivery and uptake of subcutaneously administered siRNAs to hepatocytes (Maier et al., 2013[84]). Their approach consists in using chemically stabilized siRNAs that are conjugated to tiantennary NAG (GalNAc3) on the 3' end of the sense strand of the siRNA. Hepatocyte targeting is mediated through the affinity of the ASGPR for NAG terminated oligosaccharides. Binding of the compound to the ASGPR mediates internalization via clathrin-coated vesicles; maturation of the endosome reduces the pH of the endosome which promotes dissociation of the ligand-receptor complex releasing the siRNA. The potency and stability yielded by the different chemistries used for these GalNAc3 conjugated siRNAs enables subcutaneous administration of the drug (Nair et al., 2014[97]). In addition, further stabilization of the siRNAs with an undisclosed chemical modification pattern yielded a second generation platform known as enhanced stabilization chemistry (ESC) as opposed to the standard template chemistry (STC).

#### Systemic strategies to deliver siRNAs outside the liver.

##### Kidney

Excretion of siRNAs takes place at the level of the kidney; IV administered siRNAs without a carrier fall below the filtration threshold of the kidney and are therefore rapidly taken up by the kidney and excreted in urine (van de Water et al., 2006[134]). As mentioned in the previous section, a strong signal is detected in kidney and bladder following IV administration of naked siRNAs. Studies have shown that this strong signal mainly corresponds to breakdown products of the injected compounds (Cheng et al., 2009[24]). As a consequence, chemical stabilization of the molecule has been used to improve the pharmacokinetic properties of siRNAs for renal conditions (Thompson et al., 2012[132]; Yang et al., 2014[142]). IV administration of blunt ended 19-base pair nucleotide siRNA targeting p53 with a pattern of alternate 2'-O-Me sugar modified nucleosides in both stands shows that serum stabilized siRNAs accumulate in the kidneys. Following IV administration, this compound is rapidly cleared from systemic circulation most likely due to its low affinity for plasma proteins. The highest concentration of the compound is thus observed in the kidneys shortly after administration where it is reabsorbed by the proximal tubule epithelium where the compound has a resident time below 24h. This distribution pattern correlates with the target gene knock down profile observed in kidney that is detectable already 3-6 h after administration and persists for less than 48h (Thompson et al., 2012[132]). 

##### Tumors

Solid tumors develop their own vasculature in order to cover their need in nutrients and oxygen supply. These newly formed vessels are product of aberrant angiogenesis signals that result in the formation of fenestrated and leaky blood vessels (Baluk et al., 2005[6]). In addition tumors have a poor lymphatic drainage. These structural abnormalities cause differential interstitial pressure resulting in accumulation of nanomaterials and large molecules within the tumor. This phenomenon, called enhanced permeability and retention effect (EPR), has been taken advantage of for delivering pharmaceuticals to solid tumors following intravenous administration (Seymour, 1992[119]). 

One of the first approaches designed to deliver siRNAs to tumors was by means of liposomes and LNPs (Judge et al*.*, 2009[62]; Lee et al*.*, 2010[75]). Using this approach Bisanz and coworkers (2005[13]) showed that intratumorally administered liposome encapsulated siRNAs targeting ECM-integrin were able to significantly reduce the size of bone tumors in a mouse xenograft model. Reduction of the tumors in bony tissue was accompanied by a very significant reduction in the amount of the targeted integrin. However, the dramatic decrease in tumor size was only observed when the tumor was located in the bone; this was not the case for subcutaneous tumors. Analysis of target knock-down in the subcutaneous tumors indicated that the siRNA had a very potent silencing effect, reaching levels of almost 100 %. These results point out that although the formulation was able to reach subcutaneous tumors, knocking down the target gene was not an effective mechanism to control tumor growth at this site. Similar formulations carrying a siRNA designed to silence CSN5 were effective in reducing the size of hepatic tumors following IV administration in an orthotopic mouse model of hepatocarcinoma (Lee et al., 2011[76]). Clinical validation of this approach was performed using LNPs loaded with a combination of two siRNAs, one targeting Vascular Growth Endothelial Factor-A (VEGF-A) and the other targeting Kinesin Spindle Protein (KSP). This compound, administered as an IV infusion every 2 weeks to advanced cancer patients with liver involvement, localized preferentially to the liver and spleen due to the fenestration of the capillaries in these organs. The analysis of the tumors indicated that both siRNAs were detected in the tumors with the exception of KSP siRNA that was below the LLOQ in one tumor biopsy out of twelve. The assessment of efficacy in this phase I study was complicated due to the heterogeneity of the types of tumors and to the small number of patients. 37 patients were evaluated for tumor response out of which one, suffering from an endometrial tumor with multiple hepatic metastases, showed a complete response and three others had stable disease (Tabernero et al., 2013[130]). 

##### Vascular endothelium

Formulations composed of a highly charged cationic lipid (AtuFECT01), neutral helper lipids, 1% PEG and a 2'-O-methyl stabilized siRNA, named lipoplexes, were found to differentially deliver their siRNA cargo into the endothelial vasculature of several organs following IV administration in animal models (Santel et al., 2006[116]). The goal when developing this formulation was to increase the number of positive charges per cationic lipid to allow more efficient binding of the siRNA to the lipid. In addition 1 % PEG was added in order to decrease the unspecific toxicity of cationic lipids. Studies performed with fluorescently labeled siRNAs to analyze the cellular uptake indicated that naked compounds, without any transfection agent, were actively taken up by cells when added at very high concentrations (10 µM) whereas equivalent intracellular concentrations where achieved when the formulation was added at siRNA concentrations that were approximately 1000-fold lower. Further studies showed that the intracellular distribution of the naked siRNA differed from that of the formulated siRNA; the naked siRNA was trapped in late endosomal/lysosomal vesicles whereas the formulated siRNA showed an enhanced release from endosomes reaching the cytoplasm (Santel et al., 2006[116]). The rationale behind the specific tissue biodistribution of this formulation is not fully elucidated yet. The size of the lipoplex, approximately 120 nm, seems to enable crossing of the endothelial cell membrane but impede crossing through the underlying basal membrane. In addition the increased positive charge of the lipoplex could increase uptake by endothelial cells. It should be mentioned that although several of the characteristics of the formulation increase the rate of cellular uptake the silencing efficacy seems to be tissue dependent. As such, lipoplex formulations are able to deliver and mediate gene silencing in the endothelia of some tissues i.e lung, liver, heart but are not able to so in the endothelia of other tissues such as kidney and spleen. 

### Strategies used for local delivery

The pharmaceutical properties of siRNA based therapeutics designed to have local action are different from those required by molecules designed for systemic administration. The stability of locally applied molecules needs to withstand the particular characteristics of its site of action rather than those of serum. Furthermore, reduced stability in serum can be considered an advantage for local indications in order to reduce unintended systemic side effects that may arise due to presence of the compound in systemic circulation. Size and other physical properties of the molecule/carrier system condition the entrance and clearance from the local site. Because systemic effects are unwanted for locally administered products increasing the size of the compound with a carrier is not usually required for these particular indications because avoidance of kidney clearance is not specifically needed. Size does also condition the ability of a given compound to enter the target organ. Most locally accessible tissues such as the skin or the eye are extremely well protected from external aggressions; this protection usually entails a thick physical barrier and different kinds of chemical barriers. Different strategies can be used to overcome the above mentioned barriers and deliver compounds locally. Physical approaches to deliver siRNAs across the stratum corneum barrier include microneedles (Chong et al., 2013[26]), intradermal injection (Leachman et al., 2010[73]), electroporation (Nakai et al., 2007[98]), iontophoresis (Kigasawa et al., 2010[66]) among others. Modifications of the molecule and/or formulation can also enable the molecule to penetrate into the required region and improve cellular uptake. 

#### Skin

A combination of dissolvable microneedles and a modified siRNA has been used to overcome the stratum corneum of the skin in order to improve cellular uptake. This approach has been used to obtain a proof-of-concept study on the possibility of silencing a gene in the epidermis (Lara et al., 2012[71]). CD44 is uniformly expressed throughout the five layers of the epidermis and is therefore considered an excellent target to obtain a proof-of-concept study to assess the efficacy of a delivery system to the skin. Previous human studies indicated that doses ranging between 3-17 mg of an unmodified siRNA targeting keratin 6a N171K mutant mRNA were required to observe a clinical effect in a patient of pachyonychia congenita (Leachman et al., 2010[73]). Modification of the molecule (self-delivery technology) and changing the delivery mechanism to microneedles allowed dose reductions of 20-100-fold to treat an equivalent area (Lara et al., 2012[71]). It should be noted that comparisons were made between studies that targeted different genes and these genes had different pattern of expression; therefore further studies should be performed in order to determine the specific differences between the methods of delivery. 

#### Eye

Most programs developing siRNAs for eye conditions seek modifying gene expression in the retina. For this purpose siRNAs are intravitreally injected, thus administered very close to their site of action. In this scenario strategies to improve siRNA design have been focused on increasing cellular uptake and half-life in vitreous humor. 

Self-delivery-rxRNAs (sd-rxRNAs) incorporate modifications to the molecule precisely to achieve the above mentioned goals. The specific design of these compounds consist of a 19 nt antisense strand and a shorter sense strand (<15 nt); this results in an asymmetric duplex that includes a single strand tail that is usually phosphorothioated. In addition, the strands include stabilizing 2'-F and 2-'O-Me modifications and a sterol conjugate on the SS to improve cellular uptake (Byrne et al*.*, 2013[21]). Following addition of these compounds prepared with a fluorescently labeled siRNA to cell cultures without transfection reagent indicated that the compounds were readily taken up by cells. These compounds were also able to induce target knock-down in a series of different cell types *in vitro*. *In vivo* analysis of their activity indicated that the structure and pattern of modification of these compounds enables uptake by retinal cells and ensures even distribution throughout the mouse retina. According to these *in vivo* analyses the diffusion of the compound throughout the vitreous humor and uptake by the retina is a relatively slow process. Analysis of the biodistribution of the compound 2-3 h after injection showed that the compound was only present in the ganglion cell layer at that time-point and uniform staining of the retina was not observed until 24h after injection (Byrne et al., 2013[21]). The presence of the compound in the mouse retina correlated with a target reduction of approximately 50 % 2 days after injection; the silencing effect diminished over time but was still statistically significant 14 days after injection. 

A different approach was pursued by Ahmed and coworkers (2011[1]); their results indicate that, in contrast to those of Byrne and coworkers (2013[21]), chemical stabilization of the siRNA is sufficient to enable cellular uptake and gene silencing via an RNAi mediated mechanism in retina. QPI-1007 is a 19-nt blunt ended siRNA that has alternate 2'-O-Me modified RNA nucleosides on the antisense strand, an L-DNA cytidine nucleotide on the 3' end and an inverted deoxyabasic moiety on the 5' end of the sense stand. Intravitreal injections in rats of a fluorescent siRNA with an equivalent pattern of modifications to that of QPI-1007 showed that the compound was efficiently taken up by cells. Five hours after injection approximately 90 % of retinal cells had incorporated the siRNA and the fluorescent signal was detected for up to 24 h. Further experiments performed with QPI-1007 showed that siRNA was detected by “Stem & Loop” qPCR in the retina for up to 28 days after injection (Ahmed et al*.*, 2011[1]).

The apparent contradiction of the results of Byrne et al. (2013[21]) and Ahmed et al. (2011[1]) may be rooted in differences in the experimental conditions used in their respective studies. Direct visualization of uptake using fluorescently labeled siRNA has been widely used for localization studies. However, care should be taken when interpreting these results as presence of the label does not necessarily entail presence of the intact siRNA. In addition subcellular localization is not always visualized when using labeled siRNAs, therefore additional measures should be taken to complement these analyses. Furthermore, the dose administered and timing of tissue collection are crucial factors for determining the extent of tissue distribution. On top of this, the pattern of modification of the molecule as well as the half-life and abundance of the targeted mRNA may have a direct influence on the kinetics of the subsequent processes leading to gene silencing; therefore care should be taken when extrapolating results obtained with one molecule to other siRNAs. 

## Efficacy

Gene-silencing efficacy varies significantly depending on the target gene and specific sequence chosen (Holen et al., 2002[56]). Factors influencing the efficacy of a particular approach may be related to the biology of the gene or related to the design of the siRNA. Information on the abundance and half-life of a given mRNA as well as the turnover rate of the corresponding protein influences target selection. mRNAs with a higher turnover are usually more resistant to be silenced than those with a lower turnover; the same holds true for the stability of the protein (Larsson et al*.*, 2010[72]). Methods for identifying active siRNA sequences have significantly evolved leading to the development of algorithms that now include analysis of potential secondary structures, predictions of thermodynamic stability, analysis of base composition and presence or absence of specific motifs. The correlation between the GC content and siRNA potency has been addressed by several research groups (Reynolds et al., 2004[111]; Patel et al., 2006[105]; Pei and Tuschl, 2006[107]). The results of these studies have shown that a high GC content may be a negative determinant of the functionality of a siRNA as it may inhibit the dissociation of the duplex impairing RISC loading. On the other hand, low GC content is associated with a decrease in potency, presumably due to decreased target affinity and specificity (Pei and Tuschl, 2006[107]). Recent studies have shown that siRNA sequences with a GC content in the range of 25-55 % have an increased potency (Liu et al., 2012[81]). Inclusion or absence of certain motifs has also found to have an impact on siRNA potency; as such the following motifs (5' to 3') “CUU”, “CU”, “UCU” and “GUU” have been found to have a positive effect on potency. The effect of the “UCU” motif seems to be particularly effective when positioned at the end of the guide strand (Klingelhoefer et al., 2009[68]). On the other hand, the motif “CCG” reportedly increases siRNA potency when placed at NT4-6, NT14-16 and NT16-18 (Liu et al., 2012[81]). Other motifs seem to negatively correlate with potency; an example of this in the tetranucleotide motif “ACGA”. The thermodynamic properties have also a critical role in siRNA design, thus siRNA with high potency tend to have guide strands with less stable 5'-ends than their 3' end (Reynolds et al., 2004[111]). Finally, there is a negative correlation between the self-folding tendency of a given siRNA and its potency, as the presence of secondary structures may affect the interaction between the guide strand and its target (Klingelhoefer et al., 2009[68]; Liu et al., 2012[81]) 

## Safety

siRNAs are chemically synthetized molecules that frequently contain chemical modifications that do not necessarily occur naturally in biological organisms. As mentioned previously, siRNAs are regulated as NCEs. However, the mechanism of action these molecules entails using a biological path to exert their action; hence siRNAs have some attributes in common with NBEs. The toxicities observed in response to siRNAs can thus arise as a consequence of their particular chemistry and/or the chemistry of their delivery vehicle but can also be due to activation of intended or unintended biological pathways (Black et al., 1994[14]; Levin and Henry, 2008[78]). 

Taking into account the above, toxicities associated to siRNAs are usually classified into one of two categories a) hybridization dependent toxicities and b) hybridization independent toxicities. Hybridization dependent toxicities arise from the interaction of siRNAs with other oligonucleotides through Watson and Crick base-pairing. Hybridization independent toxicities of siRNAs are not related to base-pairing (Lindow et al., 2012[80]) and are typically sub classified into toxicities associated to chemical modifications, to delivery agents or to immunomodulation. In addition, siRNAs can cause toxic phenotypes associated to the appropriation of the RNAi machinery, which is required for normal cell function. Table 1(Tab. 1) summarizes the classification of toxicities according to their origin and the strategies recommended by the Oligonucleotide Safety Working Group (OSWG) to manage and test the relevance of these toxicities (Lindow et al., 2012[80]; Kornbrust et al., 2013[69]; Berman et al., 2014[10]).

Toxicities due to base-pairing can be due to exaggerated pharmacology (EP) or off-target effects (OTEs). The former is caused by inhibition of the targeted gene to an extent that causes deleterious effects; the latter are caused by direct or indirect modulation of genes that are not intentionally targeted. 

### Hybridization dependent toxicities

#### Off-target-effects (OTEs)

siRNAs can modulate unintended genes by an RNAi mechanism, induced by the SS or the AS, or by miRNA-like effects (Birmingham et al., 2006[12]; Jackson et al., 2006[59]). The hybridization properties of siRNAs based of predictable binding to other RNA sequences enable the implementation of algorithms directed towards the reduction of off-targets. In addition certain chemical modifications of the molecule can significantly reduce the number of unintended targets. As such, Jackson and colleagues (2006[59]) found that placing a 2'-O-Me group on the second position of the guide strand inhibited off target silencing due to complementarity to the seed region without significantly affecting the silencing efficacy. Other options to reduce OTEs are selective placement of LNA residues (Puri et al., 2008[110]) or incorporation of UNA residues at position 7 of the 5'-end of the passenger strand (Bramsen et al., 2010[18]). These modifications block the incorporation of the sense strand to the RISC impairing potential OTEs mediated by the sense strand. In addition, these modifications contribute to improving the pharmaceutical properties of the duplexes by increasing their stability (Sioud, 2015[122]). As general rule, modifications that block the ability of the passenger strand to enter and induce RISC mediated silencing as well as modifications that block the phosphorylation of the 5' end of the SS such as 5'-O-methylation reduce the likelihood of OTEs (Vaish et al*.*, 2011[133]). Predictability of affecting unintended targets does not necessarily warrant that the chosen sequence does not have OTEs but enables reduction and identification of these hypothetically modulated genes. Following the identification of a potential OTEs, the relevance of the alleged off-target can be assessed by complementing the *in silico* data with relevant biological data such as effect of genetic deletions, pharmacological modulation or pattern of expression in relevant tissues. Thereafter *in vitro* and *in vivo* experiments can be set up in order to evaluate the relevance of the potential off-target for the specific therapeutic purpose of the molecule. 

#### Exaggerated Pharmacology (EP)

Modulation of gene expression by siRNAs can very efficiently change the disease state but if the pharmacological effect is greater than desired the very advantages of this drug class can be responsible for causing deleterious effects. In the RNAi field the extent and relevance of exaggerated pharmacology is dependent on the selected target gene. As such, exaggerated pharmacology is completely irrelevant for compounds targeting exogenous sequences such as viral genes; but can be relevant when targeting host genes. Relevance of EP should be addressed taking into account available data for the target gene: pattern of expression, biological consequences of deletion and/or modulation using genetic or pharmacologic tools and specific elements of the developmental program and selected candidate such as potency, administration route, dosing frequency and acceptable levels of toxicity (Kornbrust et al., 2013[69]). In addition knowledge of the distribution of the compound should contribute to investigating the relevance of exaggerated pharmacology. Strategies to restrict access of a given compound to organs were the action of the compound is not intended can be developed in order to diminish the off-site pharmacological effect. Administration of naked compounds to the eye is one example of these kind of strategies as it restricts the activity of the molecule to the intended organ by ensuring degradation when the compound reaches systemic circulation (Martinez et al*.*, 2014[88]). Other examples of modulating biodistribution to reduce unintended pharmacological effects include targeted- delivery strategies to preferentially direct a compound to the intended organ; thus avoiding long residence time in other organs. 

### Hybridization dependent toxicities

#### Toxicity related to chemical modifications or to the delivery vehicle 

Most of the toxicities related to siRNAs are linked to either its chemistry or to the characteristics of the delivery vehicle (Levin and Henry, 2008[78]). Modifications have become a common tool to increase half-life of siRNAs in biological fluids, to reduce their immunostimulatory properties and to improve other pharmacological characteristics of siRNA such as distribution, specificity and activity but it should be kept in mind that modifications affect both the efficacy and toxicity of the molecule. Most chemical modifications incorporated into siRNAs have been adopted from observations made with “their older brothers” antisense oligonucleotides. The relevance of these modifications when introduced to siRNAs is not necessarily applicable to siRNAs as dosing regimens and plasma/tissue concentration may be significantly different from those of ASOs. The following section summarizes the toxicities associated to those modifications that have been introduced to siRNAs that are currently advancing through clinical trials and of which some experience has been accumulated. 

As seen in previous section “Modification of the phosphodiester backbone”, backbone modifications are widely used to reduce nuclease activity; the most commonly used of these modifications is the replacement of a non-bridging oxygen on the backbone between two ribonucleotides with a sulphur to create a PS linkage. This modification not only enhances stability but also increases cellular uptake (Eckstein, 2014[39]). Phosphophorotioated oligonucleotides are stickier than non phosphorothioated versions and tend to strongly bind to proteins. Upon systemic administration, phosphorothioated oligonucleotides bind to plasma proteins increasing the mean half-life approximately 10 times (Eckstein, 2014[39]). Due to this ability to interact with plasma proteins phosphorothioated oligonucleotides are able to dose-dependently activate the complement cascade via the alternative pathway (Galbraith et al*.*, 1994[47]). Activation of the complement cascade leads to acute hematologic and hemodynamic disturbances that can potentially be life-threatening (Henry et al*.*, 2002[52]). These observations, made with ASOs, are dose-dependent and can be managed by slowing down the infusion rates thus keeping the phosphorotioated oligonucleotide concentrations below the threshold for complement activation (Henry et al*.*, 1997[53]). Phosphorothioated siRNAs can thus potentially also activate the complement cascade but because the concentration required for therapeutic purposes is in most cases significantly lower than that of ASOs the likelihood of these side-effect is reduced. Assessment of phosphorothioate-associated toxicities in programs developing a siRNA that reaches systemic circulation should therefore include measurement of split-products of resulting from complement activation such as C3a, C5a and Bb and assessment of blood pressure and electrocardiogram in toxicity studies and transient inhibitions of the clotting cascade due to secondary activation of neutrophils in non-human primates (NHP) (Henry et al*.*, 2014[54]). 

LNAs are formed by linking 2' and 4' carbons of the ribose ring by a methylene bridge (Koshkin and Wengel, 1998[70]). The bridge between the two carbons constrains the ribose ring rendering a structure close to that of RNA after hybridization. The constraining of the ribose increases the melting temperature of the oligonucleotide 2-8 °C per LNA nucleotide incorporated; thus enhancing the affinity of LNA oligonucleotides for complementary sequences. LNA-containing RNA duplexes are hence stabilized against nucleases (Stein et al*.*, 2010[127]). In addition, incorporation of LNA nucleotides improves activity and specificity by ensuring preferential loading of the antisense strand into the RISC (Elmen et al., 2005[42]) and may reduce immunogenicity (Goodchild et al., 2009[49]). However, introduction of these modifications in certain trinucleotide sequence motifs can increase hepatotoxicity of LNA-bearing oligonucleotides. The mechanism by which LNA-ASOs induce hepatotoxicity is not fully elucidated yet but could be initiated by the ability of these motifs to bind to hepatocellular proteins and mediated by activation of stress pathways (Burdick et al., 2014[19]). 

Chemical substitution at the 2'-hydroxyl group of the sugar moiety 2'-O-Me, 2'-F or 2'-MOE are extensively used to improve stability and reduce immunogenicity. These modifications increase the melting temperature of the oligonucleotide carrying them rendering more stable molecules and reduce immunostimulatory effects (Chiu and Rana, 2003[25]). The breakdown of products containing modified nucleotides renders however non-naturally occurring mononucleotides. These non-naturally occurring mononucleotides can potentially be incorporated into nucleotide pools and be reused for synthesis of other macromolecules. Incorporation of these modified nucleotides to newly synthetized DNA could potentially lead to genotoxicity. In addition, it is well known that certain base analogues such as fialuridine (FIAU) can cause multisystem failure due to mitochondrial damage (McKenzie et al., 1995[91]). In humans FIAU is transported into the mitochondria by the human equilibrative nucleoside transporter 1 (hENT1) and thereafter is metabolized by the thymidine kinase 2 to yield FIAU-triphosphate (FIAU-TP). FIAU-TP is a potent inhibitor of mitochondrial DNA polymerase-γ, thus causes depletion of mitochondrial DNA and cell death (Colacino et al., 1996[31]; Lewis et al., 1996[79]). It should be noted that the mouse equivalent to hENT1 is not expressed in mouse liver; therefore woodchucks have traditionally been used to assess toxicities similar to that of FIAU (Lee et al., 2006[74]). Other analogues, currently used as antivirals for treatment of HIV, act as chain terminators to suppress viral replication as they lack a 3'-OH (Johnson et al*.*, 2001[61]). These analogues can also act as substrates for the mitochondrial DNA polymerase-γ resulting in inhibition of mitochondrial replication. The results of several programs developing modified oligonucleotides for therapeutic purposes have shown that modifications studied up to date do not alter nucleotide pools nor are they genotoxic (Levin and Henry, 2008[78]). In the case of phosphorothioated oligonucleotides the European Medicines Agency specifically used available data up to 2005 to publish a reflection paper indicating that assessing the mutagenic potential of phosphorothioated oligonucleotides is not necessary (European Medicines Agency, 2005[43]). 

Finally, compounds using lipid based vehicles such as LNPs can cause infusion-related reactions, elevation of cytokines and activation of the complement cascade (Barros and Gollob, 2012[7]). Studies with several compounds using different types and composition of LNPs have shown different degree of infusion reactions; these reactions were particularly relevant when using first generation LNPs; newer versions of LNPs induce a lower immunogenic response. Infusion reactions are in general controlled by decreasing the infusion rate but in some cases pretreatment with dexamethasone and/or antihistamines is required (Tabernero et al., 2013[130]).

#### Immunomodulation

Cells have the ability to defend themselves against the entrance of exogenous genetic material. This essential function in defense against infection becomes a hurdle trying to artificially introduce an oligonucleotide for therapeutic purposes. The recognition of foreign DNA or RNA by the innate immune system induces the production of type I interferon and pro-inflammatory cytokines. The innate immune has the ability of recognizing pathogen associated material by means of germ-line pattern recognitions receptors (PPR) that promote rapid responses to microbial pathogens during the invading phase. These receptors recognize conserved pathogen-associated molecular patterns (PAMPs) which are not present in the host and are usually important for pathogenicity and/or survival of the pathogens (Parkin and Cohen, 2001[104]). PAMPs are unique to microorganisms and can be of different nature including lipopolysaccharides, peptidoglycans, capsular structures, bacterial flagellins, bacterial DNA, lipids, viral RNAs and glycoproteins. There are several proteins that specialize in recognizing PAMPS, focus in this review will be put on those proteins specializing in sensing genetic material. Proteins specialized in recognizing genetic material are present in the cytosol, and use different mechanisms to sense potentially pathogenic genetic material and mediate an aggressive response that generally leads to cell death. These enzymes include dsRNA-dependent protein kinase (PKR), 2-5 oligoadenylate synthetase (OAS), retinoic acid inducible gene (RIG-1) and melanoma differentiation-associated gene 5 (MDA-5). PKR phosphorylates serine and threonine residues of specific proteins following activation upon recognizing and binding to dsRNA. The PKR-induced phosphorylation of a certain subset of proteins leads to translation arrest and ultimately to apoptosis (Meurs et al*.*, 1990[92]). 2-5-oligoadenylate synthetase (OAS) is upregulated in response to long dsRNA and type I interferon. Activation of this enzyme results in formation of 2'-to 5'-linked oligoadenilates that subsequently activates RNAse L; enzyme that degrades cellular and viral RNAs (Samuel, 2001[113]). RIG-1 encodes a multimeric protein with two caspase recruiting domains (CARD) and a helicase domain. The helicase domain of the protein has the ability to bind to ss-RNA or ds-RNA. Upon binding, RIG-1 suffers a conformational change that allows the CARDs to interact with another CARD-containing protein named as IPS-1. IPS-1 is in turn responsible for the activation of IRF3 that results in an increase in the production of interferon β and inflammatory mediators and activation of programmed cell death program of the cell (Marques and Williams, 2005[86]; Marques et al*.*, 2006[85]). MDA-5 is structurally similar to RIG-1 but undergoes a different molecular pathway to activation probably triggered by a different subset of virus than that of RIG-1 (Kato et al., 2006[65]; Zheng et al., 2015[145]). 

Toll Like Receptors (TLRs) are mainly expressed in immune cells and are crucial for detection of pathogen-derived products and activation of immunity (Takeda and Akira, 2005[131]; Sioud, 2006[121]). TLRs are present in the cell plasma membrane where they sense external PAMPs or in the intracellular compartments where they detect foreign nucleic acids resulting from the degradation of pathogens within the endocytic compartments. TLR3, TLR7, TLR8 and TLR9 sense nucleic acids; TLR7 and TLR8 recognize ssRNA rich in guanosine and uridine (Hornung et al*.*, 2008[57]). Activation of TLR8 seems to be more sequence specific whereas it is thought that a ribose backbone and multiple uridines are required for activation of TLR7 (Judge et al*.*, 2005[63]; Diebold et al*.*, 2006[36]). It should be noted that although mice express TLR8 this receptor is not activated by the same stimuli as it is in humans and should therefore be taking into account when assaying the immunostimaltion properties of siRNAs in preclinical developmental programs. TLR3 recognizes dsRNA and is present in intracellular compartments of mature dendritic cells and a whole wide range of non-immune cell types including epithelial, endothelial and fibroblast lines. siRNAs of 21 nucleotides or more activate this receptor inducing a cytokine response mainly composed of INFγ and IL-12 (Kleinman et al*.*, 2008[67]). 

Several strategies have been developed to enable synthetic siRNAs evading detection by the innate immune system. These strategies include design rules, chemical modifications and formulation in carriers to avoid delivering naked siRNAs into endosomes. A wide variety of chemical modifications that, as discussed in section “Stability of RNAi based therapeutics: chemical modification” confer nuclease resistance have been successfully used to abrogate siRNA immune activation. Morrisey and colleagues showed that the incorporation of 2' modified nucleotides such as, DNA bases, 2'-O-Me purines, 2'F pyrimidines, terminal inverted-dT bases, and PS linkage at selected positions can abrogate siRNA immune activation (Sioud, 2006[123]). As mentioned previously, chemical modification of the molecule regardless of the final aim should be handled with care in order to avoid loss of silencing activity. Replacement of uridines with their 2´Fluoro, 2'-deoxy or 2'-O-Me modified counterparts can block TLR recognition without significantly reducing the silencing potency (Sioud, 2006[123]). The same holds true for the replacement of uridines for thymidines in the sense strand (Flatekval and Sioud, 2009[46]). Ideally, *in vitro* studies should be performed using a mixed population of primary immune cells such as fresh peripheral blood mononuclear cells to test immunogenicity in vitro, but most importantly, signs of immune activation should be monitored *in vivo* by examining cytokine levels and activation of inflammatory pathway genes at a short time-point and 24 hours after administration (Coch et al*.*, 2013[28]).

## Programs in Clinical Development

With two compounds in phase III and several compounds in advanced phase II the RNAi filed is certainly getting closer to the market. Table 2(Tab. 2) summarizes the current status of clinical candidates and the characteristics of each compound. 

### Products using systemic administration

#### Targeting the liver

##### Transthyretin amyloidosis

Patisiran (ALN-TTR02) targets transthyretin (TTR) and is the most advanced candidate in the RNAi pipeline (phase III) (Coelho et al*.*, 2013[29]). This compound, being developed for the treatment of familial amyloidotic polyneuropathy (FAP), obtained orphan drug status in US/EU and fast track designation by the FDA. FAP is an autosomal dominant condition caused by mutations to the TTR gene. FAP causing mutations in the TTR gene yield defective proteins that fail multimerize to form the functional tetramer and instead self-assemble into amyloid fibrils that subsequently accumulate in different organs (Hou et al*.*, 2007[58]). Most individuals carrying mutations in the TTR gene are heterozygous; thus accumulations found in tissues of affected individuals contain both mutant and non-mutant protein. Accumulation of liver derived TTR in the peripheral nervous system is thought to be the primary cause of FAP although the neurotoxic mechanism induced by TTR accumulation is poorly understood. TTR is present in plasma and cerebrospinal fluid where it is involved in the transportation of thyroxine and retinol but the role of this protein does not seem to be essential for life. As such, reduction of TTR appears to be a valid approach to treat the disease. Patisiran is a siRNA designed to silence non-mutant and mutant TTR encapsulated in a LNP formulation. Human trials for this compound have shown a robust, dose-dependent and durable reduction of the targeted protein in serum in response to single and repeated doses of the compound (Coelho et al., 2013[29]). Six-month clinical data of a phase II extension study in which patients received infusions of 0.3 mg/kg every three weeks were recently presented and indicated that the compound possibly stabilizes disease progression. The main side-effects observed in response to patisiran were infusion-site reaction (14.8 %) and flushing (14.8 %) most likely caused by the delivery vehicle.

Revusiran (ALN-TTRsc) is an adaptation of patisiran into the new NAC-based delivery platform. This compound is currently in phase II for another type of TTR-amyloidosis: familial amyloidotic cardiopathy (FAC). In this type of amyloidosis cardiac amyloid deposition leads to cardiac wall thickening and heart failure (Hermansen et al*.*, 1995[55]). The change in delivery method of this compound allows subcutaneous administration which also requires change of dose regimen. In the ongoing phase 2 study daily doses of 5 or 7.5 mg/kg are given during an initial loading period of 5 days and thereafter weekly doses are administered until the end of the study. The main side effects observed up to date in response to revusiran are injection site erythema (23 %) and transient elevation of liver function tests (15 %). The maximum serum TTR knock-down observed in this study up to date is 98.2 % with a mean maximum knockdown of 87 %. 

##### Cardio-Metabolic diseases

ALN-PCS02 is a LNP formulated siRNA targeting proprotein convertase subtilisin/kexin type 9 (PCSK9), a key protein in the regulation of cholesterol homeostasis. PCSK9 enhances degradation of hepatic LDLR, thus resulting reduced hepatic LDL-cholesterol clearance. Loss of function mutations in PCSK9 have been described in humans and are associated with reduced LDL cholesterol and coronary heart disease (Cohen et al*.*, 2006[30]). A phase 1 trial was performed with ALN-PCS02 in subjects with cholesterol levels above 3.00 mmol/L. The results of this trial showed that a single dose of IV infused ALN-PCS02 was well tolerated and the highest doses tested caused a significant reduction in plasma PCSK9 and serum LDL-cholesterol. Patients in this trial were pretreated with immunosuppressants and antihistamines to avoid a potential immune stimulation response associated with LNP based delivery vehicles (Fitzgerald et al., 2013[45]). Further development of this program will be performed with ALN-PCSsc, a compound co-developed by Alnylam and the Medicines Company that entails a change into the NAC-based delivery platform and thus subcutaneous (SQ) dosing. A phase I study has recently been initiated for this compound with the goal of assessing safety and tolerability of single ascending doses as well as multiple dosing in patients with elevated baseline LDL-C. This compound also incorporates improvements to the NAC-based technology that are the basis for the ESC-technology (Nair et al., 2014[97]).

TMK-ApoB was one of the first compounds based on RNAi to reach clinical trials. The program, terminated in 2010, used a siRNA targeting Apoliprotein B (ApoB) encapsulated in first generation LNPs. The phase I clinical trial was halted due to unexpected immune stimulation. The preclinical evaluation of this product had not anticipated the subsequent events observed in the phase I trial thus raising the question whether preclinical models were good predictors of the immune response. Much has been learnt since the mentioned trial and new formulations have been designed in order to reduce the immunostimulatory properties of LNPs; this has been partially achieved by modifying the composition of LNP as well by reducing the infusion rates while administering the compound (Coelho et al., 2013[29]). In addition mechanistic studies have shed light on the underlying causes for the LNP-induced immune stimulation and more importantly the failure in preclinical models to anticipate the mentioned toxicities (Coch et al., 2013[28]). 

##### Liver cirrhosis and fibrotic disease of the liver

ND L02-s0201 is a siRNA targeting Heat Shock Protein 47 (HSP47) formulated in vitamin A-conjugated LNPs under development by Nitto Denko for the treatment of fibrotic diseases of the liver (Sato et al*.*, 2008[117]). HSP47 is an endoplasmic reticulum chaperone specific for the correct folding and stabilization of procollagen (Nagata, 1996[96]). Inhibition of this chaperone impairs collagen maturation and has been shown to be beneficial in liver fibrosis (Arthur, 2002[5]). The vitamin A moiety provides receptor mediated targeting to hepatic stellar cells by the affinity of vitamin A for retinol binding protein. This product is currently undergoing phase Ib/II clinical trials in patients with moderate to extensive liver fibrosis that are receiving either a once a week or a twice a week IV dosing of the compound. 

##### Cancer

TKM-PKL1 is a LNP formulated RNAi trigger that targets polo kinase 1 (PLK1). PLKs are a family of proteins with important roles in cell cycle regulation. PLK1 can override certain regulation points in the cell cycle causing genetic instability and promoting tumor development (Strebhardt and Ullrich, 2006[128]). One phase I trial for primary or secondary liver cancer has been completed for this compound and two phase 1/2 trials, one for hepatocellular carcinoma and the other for neuroendocrine tumors, are currently ongoing. TKM-PLK1 is formulated in first generation LNPs and is therefore able to induce immunogenic responses. Cytokine release symptoms occurred at the limit-dose of 0.9 mg/Kg, dose at which 25% of the patients showed elevations 6 h after infusion. This elevation in cytokines required additional immunosuppression (dexamethasone and diphenhydramine 3 hours after dosing) on top of the pre-dose immunosuppression. 

Atu027 is Silence Therapeutic's clinical candidate for cancer. The product is a modified siRNA targeting Protein Kinase N3 (PKN3) formulated in lipoplex nanoparticles. The particular lipid composition of the formulation facilitates delivery to the vascular endothelium. PKN3 is a downstream effector of phosphatidylinositol 3-kinase (PI3K), a kinase that controls a transduction pathway commonly dysregulated in several forms of cancer. Silencing PKN3 stabilizes the vasculature and attenuates inflammatory responses in tumors reducing vasculature leakage and permeability; as a consequence there is a reduction in extravasation of metastatic cells and a reduction in the number of metastases in animal models (Santel et al., 2006[116]). A phase I dose escalating study was performed with this drug in patients with different types of solid tumors; the results of this study showed that the drug was well tolerated and disease stabilization over a period of 8 weeks was observed in 41 % of the patients. 

#### Kidney disorders

Initial biodistribution studies showed that systemically administered siRNAs are excreted through the kidney, thus it seemed logical to develop compounds to treat conditions related to a tissue that is seemingly exposed to these compounds following IV administration. QPI-1002 is a naked siRNA modified with alternate 2'-O-Me on both stands targeting p53 under development by Quark Pharmaceuticals. This compound has completed a phase I clinical study for kidney injury in acute renal failure and a phase II for the prophylaxis of delayed graft function in deceased donor kidney transplant patients. This phase II trial finished in 2014 and failed to meet its primary endpoint which aimed a 30 % relative risk reduction compared to placebo. The sponsor, however highlighted, that this endpoint was indeed met in a certain set of the population. The company is planning a phase III trial to start in 2015. 

#### Viral infections

In terms of safety, targeting an exogenous genome, completely different from that of the host organism seems an ideal situation. Off-target effects are theoretically greatly reduced and issues with exaggerated pharmacology are not relevant.

With this in mind several companies are developing compounds that target viral genomes. The most advanced of these compounds is Arrowhead Corporation's ARC520 (Wooddell et al., 2013[140]). This compound formulated as a two-molecule DPC administered by the IV route targets the hepatitis B virus (HBV) genome. The formulation is composed of a combination of two chemically stabilized siRNAs conjugated to cholesterol and a masked hepatocyte targeted peptide that promotes endosomal escape. The targeting moiety of the peptide is a NAG as overviewed in the “delivery” section of this review. The goal of this drug is to achieve a functional cure of HBV that is an immune clearant state characterized by HBsAg-negative serum. The mechanism proposed to achieve the above mentioned goal is to reduce the virus replication and to induce an immune response aimed at clearing the virus present in the system. The compound was tested in a phase 1 dose-escalating study in healthy volunteers who received a single administration of doses up to 4.0 mg/kg, the results of this study showed that the compound was well tolerated with no relevant clinical findings. The compound was subsequently evaluated in a phase IIa trial in subjects with chronic HBV patients that were negative for the antigen e of HBV (HBeAG) and under treatment with entecavir, an inhibitor of viral replication. The experimental treatment was administered as a single dose of one four doses or placebo (1.0-4.0 mg/ kg); the patients continued on a daily dose of entecavir and received an oral dose of antihistamines prior to administration of ARC520 or placebo to prevent possible skin reactions. The interim results of this trial indicated that there was a reduction in the HBsAg of 39 % in response to the dose of 1.0 mg/kg and a 57 % reduction in HBsAg in response to the dose of 2.0 mg/kg. The compound was well tolerated at both doses. The reduction in the HBsAg in response to the dose of 2.0 mg/kg was however below the expectations and not sufficient to reduce the antigen to levels at which the innate immune response can clear the virus achieving a functional cure which are estimated to be approximately 90 % knock-down. The results of subsequent doses will point out if the goal set out by the company is indeed achievable of if further doses of the drug will be required to accomplish a functional cure. 

TKM-Ebola is a combination of three siRNAs that target three genes of the Zaire strain of the Ebola virus: the polymerase L, VP24 and VP35 (Geisbert et al*.*, 2010[48]). The polymerase L interacts with VP35 to generate the polymerase complex that transcribes and replicates the virus genome. VP24 is a structural protein; VP24 and VP35 also play a role in reducing the host type 1 interferon response (Sanchez et al*.*, 1993[114]). The combination of siRNAs is encapsulated in LNP and the final product is a lyophilized powder. This compound is being developed under the tenets of the animal rule. A phase I study was initiated in 2014 to assess the safety, tolerability and pharmacokinetics of escalating doses of TKM-Ebola in healthy volunteers. The single dose ascending dose portion of the study was completed (0.075-0.5 mg/ kg), but prior to starting the multiple dose part of the study the FDA issued a hold on the program to ask the company to further investigate cytokine releases observed in response to the single dose. The results of the study showed a transient elevation in MCP‐1, IL‐6, IL‐1 and IL‐8 that returned to base levels 24 h post-dosing. Further investigations indicated that this dose limiting toxicity was present in response to doses higher than 0.3 mg/kg. The clinical hold has subsequently been modified to a partial hold to allow the drug to be used in infected individuals in the current Ebola virus outbreak taking place in West Africa. 

### Programs using local administration

#### Skin

RXI-109, a self-delivery siRNA, targets Connective Tissue Growth Factor (CTGF) and is being developed for the treatment of hypertrophic scaring and keloids. Self-delivery siRNAs (sd-siRNA) are asymmetrical double stranded RNAs are thought to combine the potency of siRNA with the improved biodistribution of antisense oligonucleotides. RXI-109 is a chemically stabilized sd-siRNA that incorporates hydrophobic moieties for improved cellular uptake (Byrne et al., 2013[21]). The compound was intradermally injected in a single dose phase I trial to analyze safety a preliminary clinical effects of the compound. In this trial healthy volunteers received an intradermal injection of the compound at doses ranging from 1 to 10 mg/ incision site into two incision sites in the abdomen and placebo into two other sites. In a subsequent phase I trial multiple doses of the compound (three doses over two weeks) were injected into four incisions located on one side of the abdomen at doses ranging from 2.5 to 7.5 mg/incision and placebo was administered via the same route into four incisions on the other side of the abdomen. The compound was well tolerated in both studies and induced a dose-dependent decrease in protein and mRNA levels of CTGF. Three phase II studies were initiated in 2013-2014 to evaluate the efficacy of the compound on lower abdominal scars, keloids and hypertrophic scars. Patients of the lower abdominal scar study received the drug on a weekly schedule starting immediately after surgery (immediate group) or two weeks after surgery (delayed group). The results released by the company indicate that blind evaluators were able to identify the treated side 54 % of the time in the delayed group and only 24 % of the time in the immediate group at the 3-month follow up visit. The results in the phase IIa study of lower abdominal scar induced the company to amend the keloid and hypertrophic scar studies in order to fine-tune the treatment schedule. 

#### Eye

The eye offers several advantages as a target organ for RNAi therapeutics. It has a relatively low content in RNases, and it is quite isolated from the rest of the body, limiting the access of the compound administered into this organ to systemic circulation (Pañeda, 2012[103]). These advantages made the eye one of the first targets of RNAi therapeutics. Unfortunately reports of immune stimulation by siRNAs 21 nt or longer diminished the initial enthusiasm of RNAi based treatments for ocular conditions (Kleinman et al., 2008[67]). In the last few years, characterization of the specific immune response evoked by siRNAs and the development of specific assays to investigate this response have allowed recovering some of the initial interest. 

The most advanced programs for ocular indications are Quark's QPI-007 designed to silence Caspase 2, currently in Phase II/III for the treatment of Non-arteritic Anterior Ischemic Optic Neuropathy (NAION) and Sylentis' bamosiran (SYL040012), targeting β2-Adrenergic Receptor (ADRB2) for the treatment of glaucoma, in phase IIb. QPI-1007 is a 19-nt modified siRNA that has shown to be safe when injected intravitreally (IVT) to animal models and humans (Ahmed et al., 2011[1]; Solano et al., 2014[124]). The compound is currently being investigated in a phase II/III trial to analyze whether multiple IVT doses of this compound are able to improve visual acuity in patients suffering NAION. Bamosiran is a canonical-designed naked siRNA targeting ADRB2 (Pañeda, 2012[103], 2013[101]; Martinez et al., 2014[88]). Glaucoma is a degenerative, chronic disease of the optic nerve that can lead to blindness if left untreated (Weinreb and Khaw, 2004[137]). The mechanistic details of optic nerve degeneration observed in glaucoma are yet to be fully detailed, but it is well established that reduction of intraocular pressure avoids development of the disease. ADRB2 controls production and release of aqueous humor, the liquid that fills the eye, at the ciliary body. This liquid is responsible for providing nutrients to the cells inside the eye; it is also responsible for maintaining optimal intraocular pressure. Treatment with topic beta-blockers has shown to efficiently reduce intraocular pressure but currently approved beta-blockers are small molecules and are thus able to reach systemic circulation and systemic organs were they cause unwanted effects. The rationale behind bamosiran is developing a locally active compound that is efficient inhibiting ADRB2 in the eye but that is not able to reach systemic tissues reducing the likelihood of side effects. The compound is administered in eye drops and has been shown to be well tolerated in animal models and humans (Martinez et al., 2014[88]; Moreno-Montanes et al., 2014[93]). Several doses of bamosiran are currently being studied in an active controlled phase IIb trial. Previous clinical trials with this compound have shown promising results in healthy individuals and patients with ocular hypertension (Moreno-Montanes et al., 2014[93]; Pañeda et al., 2014[102]). 

SYL1001 is a naked 19-nt siRNA targeting transient receptor potential vanilloid-1 (TRPV1) for the treatment of ocular pain. TRPV1 is a cation channel permeable to calcium activated by stimuli such as heat, low pH and capsaicine; this receptor is present in several structures of the eye where it has been related, among other roles, to nociception (Martinez-Garcia et al., 2013[ref:90]). This compound has shown to be safe when administered in eye drops to animals models and humans and is undergoing a phase I/II for the treatment of ocular pain associated to dry eye disease, a condition for which no specific treatment currently exists (Pañeda, 2012[ref:102]).

## Steps towards Innovation

The encouraging advances observed in the clinical setting have enabled further research in the field of RNAi therapeutics. Most preclinical pipelines are taking advantage of the discoveries and lessons learned from the compounds that are currently advancing though clinical trials. Problems identified in developmental programs of both siRNAs and ASOs have been taken back to the lab in order to find innovative ways of solving them. As such, a great deal of programs in preclinical research are taking advantage of the innovations made to facilitate delivery of siRNA based compounds to the liver; but we are also starting to see new approaches to deliver siRNAs to organs outside the liver, incorporation of algorithms to reduce toxicity, instability etc. Table 3(Tab. 3) summarizes candidates that are currently advancing towards clinical validation. 

Alnylam has, in the last few years, been very active in incorporating new candidates to its preclinical pipeline; particularly candidates that target genes that require down-regulation in the liver. In the last months they have implemented their newly presented delivery platform into most of their preclinical candidates, hence shifting the delivery strategy from one based on LNPs to a NAC based delivery. Reasons for this shift include a better efficacy/toxicity ratio and a patient-friendlier administration route: subcutaneous instead in intravenous. In addition, the NAC delivery platform is fully own by Alnylam, excluding the requirement of licensing external intellectual property. Among the preclinical candidates only one, ALN-PCSsc, uses the first generation standard chemistry (STC) NAcs; whereas the newer candidates use the improved version of this technology: enhanced stabilization chemistry (ESC). Recent data have also shown that NAC conjugated siRNAs can also be administered to mice via inhalation using high pressure microsprays (Presentation at the 10^th^ OTS meeting). The biodistribution of the siRNAs and the knock-down of the target gene, monitored by measuring the serum levels of the encoded protein, appear to be equivalent to those observed in response to SQ-administration. These results open up a new avenue for needle free administration for liver targeting 

Arrowhead Corporation has recently filed a CTA for initiation of clinical trials with ACR-ATT, a compound for the treatment of alpha-1-antitrypsin deficiency. It is expected that clinical trials with this compound are also amenable to SQ administration. In addition to Alnylam and Arrowhead, Tekmira has also launched two new preclinical programs (TKM-GSD and TKM-HTG) that most likely require reduction of gene expression in the liver. According to recent press releases these new programs might pursue a multi-target approach. Multi-targeting is an obvious direction for the field, as silencing multiple targets in a given disease would most likely increase potency. As of today, multi-targeting has only been approached to tackle viral infections (TKM-Ebola, ARC520) (Geisbert et al., 2010[48]) and cancer (ALN-VSP) (Tabernero et al., 2013[130]) but the experience accumulated by taking these compounds through clinical programs will certainly boost multi-targeting approaches for other conditions. 

Delivery of compounds outside the liver is another of the challenges that the RNAi field will face in the upcoming years. A few steps towards this direction have already been taken and hopefully the fruit of this labor will start to generate strategies that can be taken into clinical trials in the next few years. Examples of this include Silence Therapeutics' Atu111, a compound that targets Angiopoietin-2. This compound uses a delivery platform of poly-lipoplexes developed by the company that has been shown to deliver RNAi triggers into the lung endothelial cells. Another of the most likely goals for this technology is achieving delivery to the central nervous system. The long-lasting effects of RNAi make this difficult-to-reach organ an ideal target for RNAi based approaches. Several receptors present in the blood-brain barrier, such as the transferrin receptor, have been used in order to develop delivery strategies for RNAi triggers into the central nervous system. These techniques need however to be refined in order to be fully exploited for therapeutic use (Boudreau et al*.*, 2011[15]). 

## Conclusion

The last decade has witnessed the remarkable evolution of the use and exploitation of RNA interference with the aim of developing therapeutics. It has been a rough path characterized by volatility in support and funding. The introduction of chemical modifications as well as the development of innovative carriers with the ability of delivering RNAi triggers closer to their site of action, have significantly improved the pharmaceutical properties of these compounds. In addition, the robust of long lasting gene knock down seen in several human trials has shown that RNA interference is in fact a very promising tool in the therapeutic landscape. The following years will be crucial to see if this technology is able to give hold up to the expectations and whether RNA interference indeed becomes a new class of medicines. 

## Acknowledgements

The authors apologize to colleagues whose relevant primary publications and ongoing research have not been cited because of space constraints but whose work has helped move the field forward. The authors would like to thank B. Vargas and L. Company for useful discussions and suggestions and for reading the review. 

## Conflict of interest

The authors are all employees and stockholders of Sylentis S.A.U., a Spanish biotechnology company focused on developing gene silencing technology based therapies.

## Figures and Tables

**Table 1:**
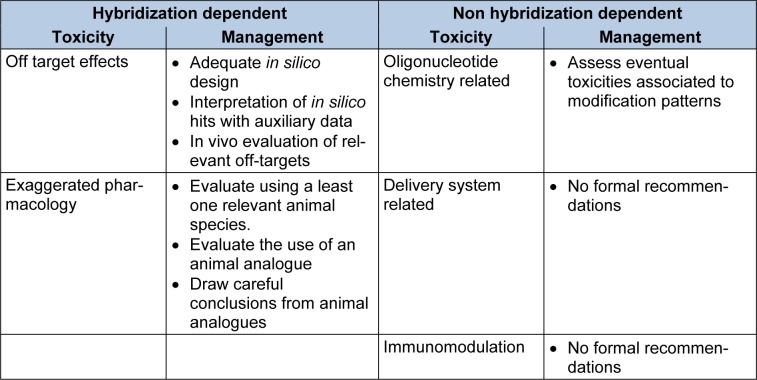
Classification of toxicities of siRNA-based compounds and recommendations to assess eventual toxicities

**Table 2:**
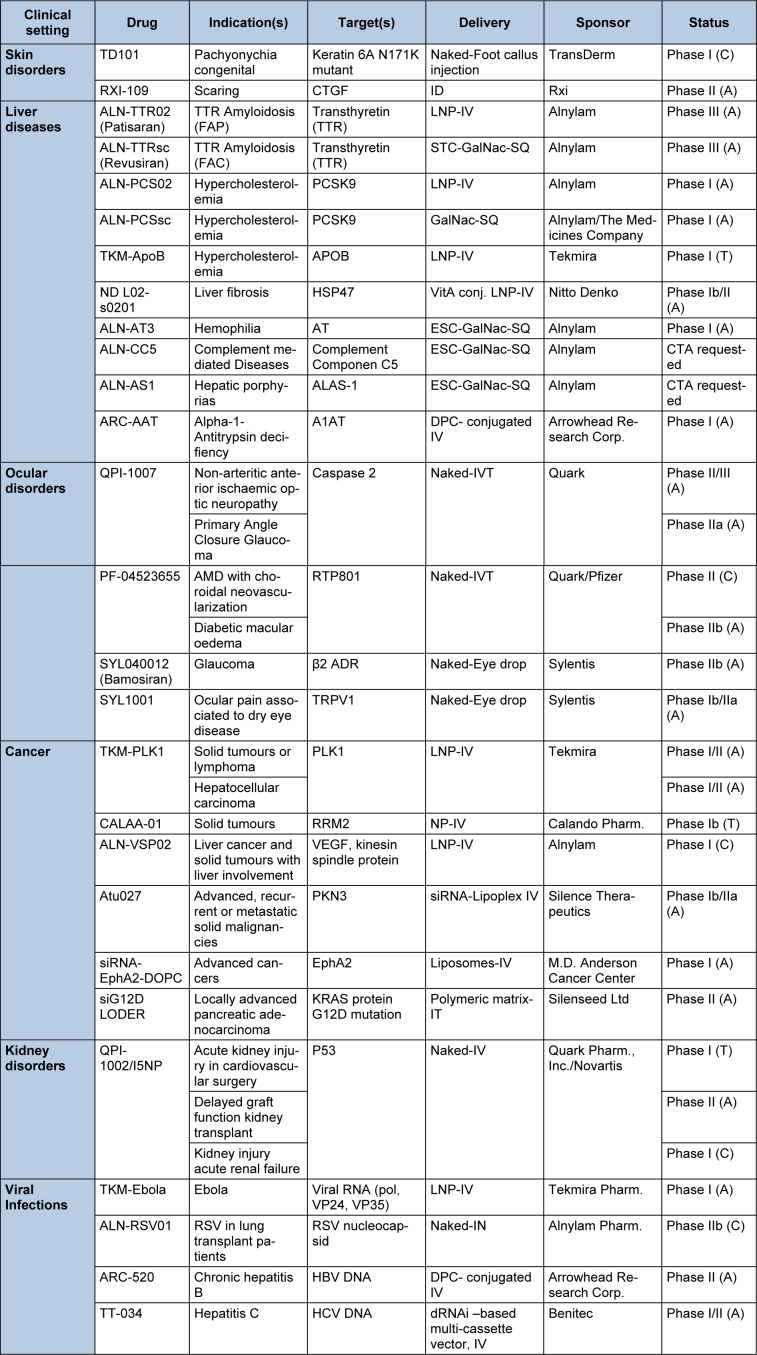
Programs in clinical development. (A) Active, (C) Complete and (T) Terminated

**Table 3:**
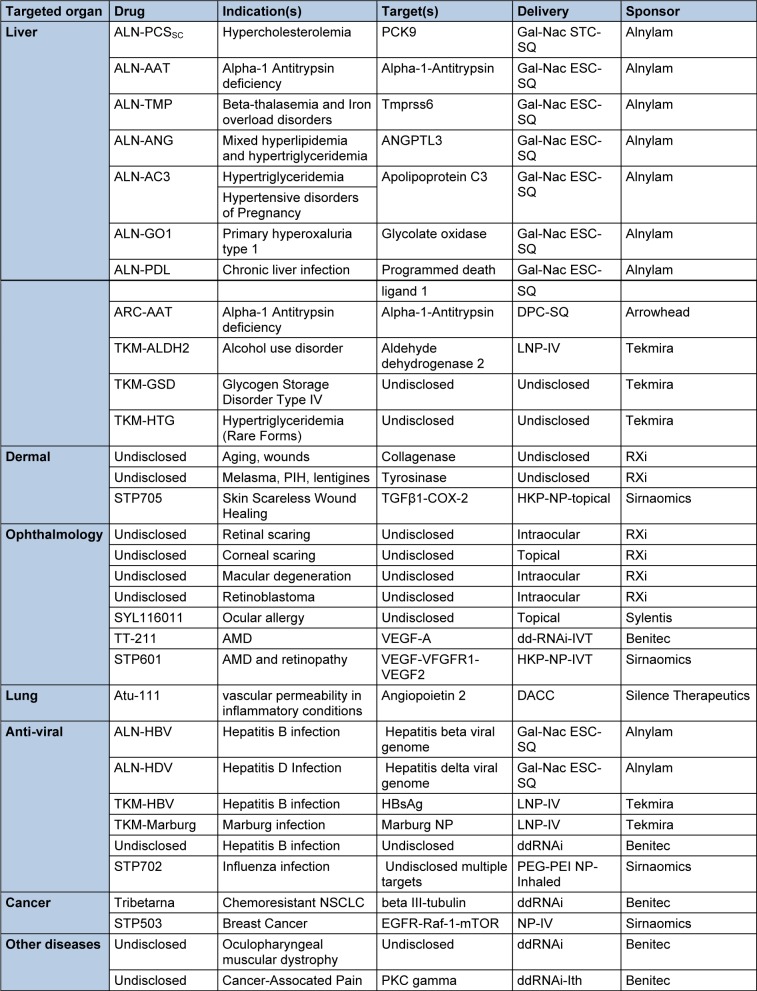
Programs in preclinical development
